# Death of Professor Gorham

**Published:** 1829

**Authors:** 


41. *Death of Professor Gorham.*—We are indebted to the Boston Medical and Surgical Journal, for an interesting account of the life and death of this very respectable physician and public teacher. He died at his residence, in Boston, on the 27th of March, 1829, after an illness of four or five days. His disease was an inflammation of the pleura and inferior lobe of the lung, of the left side. On examination after death, the surface of the pleura was found covered with coagulated lymph, and extensively agglutinated to the lung, the lower parts of which were solidified, and resembled boiled lung moderately compressed.

Dr. G. was in middle life. After graduating in the Arts, at Harvard University in 1801, he studied medicine with the elder Professor Warren. He then spent two years in Europe, and on his return married the daughter of his preceptor. Soon afterwards he was appointed adjunct to the late Dr. Dexter, professor of Chemistry in his *alma mater*; and while in this situation delivered, with approbation, several courses of popular lectures on his favorite science, to the citizens of Boston. Meanwhile he began to acquire an extensive and profitable business as a physician.

In the year 1811, his colleague having resigned his office, Doctor Gorham was chosen Professor of Chemistry and Mineralogy in the University of Cambridge, and continued to give lectures in Boston and Cambridge till 1827, when the great increase of his business led him to resign this post, and devote himself wholly to private practice. During the time of his Professorship, he published a valuable System of Chemistry, in two octavo volumes. He wrote many papers in the New England Journal of Medicine and Surgery, of which he was a joint Editor for about fifteen years, and contributed also to the Boston Medical and Surgical Journal. He lent his aid to the improvement of Medical science, by serving as Recording Secretary of the Massachusetts Medical Society, and held various other offices, which demanded labor and ability. He was to have delivered the next annual discourse of the Massachusetts Medical Society. His practice regularly increased till it became so great as to require efforts and exposures which predisposed him to disease, and threw him in the way of its exciting causes.

The remarks of the Editor on the moral and social character of the deceased, we shall transcribe for the benefit of the younger members of the profession.

Dr. Gorham had arrived at the period of life when a good physician is most valuable to the community; at the period, when to learning is added personal experience, when the judgment is mature, and when the confidence, not of a few individuals only, but of the public at large, is secured and settled. He was a good physician indeed, and the community realised his worth. He was in the midst of a full business, and ardent in the prosecution of it. He obtained not only the confidence but the love of his patients, and enjoyed at the same time the respect and good will of his professional brethren.

Justice may call on us to say so much, and would permit the use of stronger terms. For the benefit of survivors, it may be proper to inquire how he obtained so much respect, confidence, and good will. It was by dilligence and fidelity in seeking for knowledge and in using it; by rectitude in principle and urbanity of manners. But he had a charm, which these words do not describe, and which was felt by all who knew him. This charm arose from the simplicity, if it may be so termed, which marked his character. Frank and open-hearted, free from selfish propensities, he sought to make others happy, without thinking of the merit of it; and he succeeded; for his face, truly beaming with benevolence, cast a bright light on those around him, while his kind feelings begat a sympathetic good will among his friends and companions.

The Funeral ceremonies were conducted in a manner correspondent with the character of the deceased, but our limits forbid us from adding more, than that professor Jackson pronounced his eulogy, "in a simplicity and elegance of style admirably suited both to its subject and its author."

